# Risks, precipitants and clinical presentation of gastro-oesophageal reflux disease at the Kilimanjaro Christian Medical Centre in Tanzania

**DOI:** 10.11604/pamj.2014.19.119.3575

**Published:** 2014-10-01

**Authors:** Michael Bartholomew Mwandri, Julius Chacha Mwita, Mgaywa Gilbert Mjungu Damas Magafu, Kajiru Gad Kilonzo, Sarah Japhet Urasa

**Affiliations:** 1 University of Botswana, Private Bag 713, Gaborone, Botswana; 2 Kilimanjaro Christian Medical Centre, Tanzania P.O.Box 3010, Moshi, Tanzania

**Keywords:** GERD, precipitating food, risk factors, loose lower ooesophageal sphincter, upper gastrointestinal endoscopy

## Abstract

**Introduction:**

Risk factors and precipitants of gastro-oesophageal disease (GERD) differ widely in communities. We conducted an observational study to describe these risks, precipitants and clinical presentation of GERD patients at Kilimanjaro Christian Medical Centre (KCMC) in Tanzania.

**Methods:**

We consecutively recruited 92 GERD patients who were referred for endoscopy at KCMC from March to November 2008. By using structured questionnaire we enquired: risk factors, precipitants and symptoms of GERD and upper gastrointestinal endoscopic findings. Their upper gastrointestinal endoscopic findings were as well documented.

**Results:**

The mean (± SD) age of the study population was 47.32 (±17) years. Reported symptoms included water brash (37%), dyspepsia (6%), chronic cough (11%) and hemoptysis (5%). More than half (56%) of the patients surveyed identified food precipitants for their GERD symptoms. Triggers of GERD symptoms were boiled beans 19%, spicy food 11%, sour/fermented meals 10%, roasted tomato 9%, silver cyprinid fish (dagaa) 5%, beans with cooked green banana (matoke) 2% and fermented milk 1%. Most of the studied patients had normal body mass index (52%), and 25% admitted to be consuming alcohol though they didn't associate it with their GERD symptoms. The most common endoscopy finding was “loose lower oesophageal sphincter” (85%).

**Conclusion:**

Most GERD patients referred for endoscopy at KCMC were found to have water brash and “loose lower oesophageal sphincters” as described by endoscopists to denote mechanical abnormality of the lower oesophageal sphincter. GERD symptoms were precipitated by common locally available food and spices.

## Introduction

Gastro-oesophageal reflux disease (GERD is a chronic condition that affects 10-26% of the general population [1, 2]. It classically presents with heart burn, acid regurgitation and water brash at least once a week [1, 3, 4]. The above symptoms are referred to as oesophageal symptoms, and are due to backward acid flow into esophagus. In long-term, acid regurgitation may cause oesophageal ulceration, fissures, bleeding, strictures and the pre malignant Barrett's esophagus [4]. Patients may also present with extra-oesophageal symptoms such as laryngeal disorders, hoarseness, chronic cough, asthma or non-cardiac chest pain. Extra-oesophageal GERD symptoms are due to reflux of acid into the air ways [5].

Risk factors of GERD vary widely in different population worldwide [4, 6–8]. They include cigarettes smoking, obesity, alcohol consumption, mint, fatty food and drugs which interfere with resting pressure of the lower oesophageal sphincter [6, 9, 10]. Other reported risk factors are anti-inflammatory drugs, pregnancy, hypothyroidism, systemic sclerosing disorders, prolonged use of nasogastric tube and postprandial lying position [11]. Although risk factors and precipitants are often mentioned separately, in some instances they may be used interchangeably [12].

The diagnosis of GERD can be established by assessing the response to anti-secretory agents [13, 14], barium studies, endoscopy, Ph monitoring studies, and oesophageal manometry [1, 15]. Although the gold standard investigation for diagnosing incompetence of the lower oesophageal sphincter is oesophageal manometry, endoscopy can be used to assess the competence of lower oesophageal sphincter [16]. Endoscopy can as well be used to evaluate GERD complications such as Barrett's oesophagus, dysphagia, odynophagia, upper gastro intestinal bleeding, oesophageal ulcers and strictures [1].

There is paucity of information on risk factors, precipitants and clinical characteristics of patients with GERD in Africa [2]. This study aimed to describe risk factors, precipitants and clinical presentation of GERD patients referred for upper gastrointestinal endoscopy at Kilimanjaro Christian Medical Centre (KCMC).

## Methods

### Study design, setting and participants

We conducted a descriptive cross sectional study and consecutively recruited GERD patients referred to the endoscopy unit at Kilimanjaro Christian Medical Centre (KCMC) for a period of 4 months. KCMC is the major hospital in northern Tanzania and is among the 4 consultant hospitals in the country. It has 500 bed capacity and an endoscopy unit that serves 20- 30 patients per week. We excluded patients who came for follow-up after surgical intervention, those known to have other causes of regurgitation apart from GERD and those who were too sick to be interviewed or to undertake anthropometric measurements.

### Data collection

A standardized questionnaire was used to capture information from patients and from endoscopy reports. Demographic characteristics such as age and sex were documented. GERD known risk factors such as obesity, alcohol consumption and use of tobacco were recorded. Other information recorded included the consumption of risky food substances (oily meals, spicy meals, tomatoes, pepper mint and chocolate), and the use of drugs precipitating GERD symptoms such as none steroid anti-inflammatory drugs (NSAIDs), calcium-channel blockers, benzodiazepines and nitrates. Furthermore we requested patients to identify local food substances which precipitate their GERD symptoms. We enquired and documented presence of symptoms of GERD such as regurgitation, dyspepsia, heartburn, water brash, hematemesis, chronic cough, hoarseness of voice, dysphagia, odynophagia and asthma-like symptoms. We furthermore documented their anthropometric measurements and findings of the upper gastro intestinal endoscopy examination.

The study was approved by the institutional research ethics committee at the Tumaini University, Kilimanjaro Christian Medical College, Tanzania. (Ethical clearance certificate No. 239, for research proposal No. 279 titled ‘the audit of GERD at KCMC), and was conducted according to the ethical guidelines of the declaration of Helsinki. Briefing of the study to potential participants was done and consent sought before enrolment. The statistical program SPPS Version 20.0 for Windows was used in the analysis of results and the results were expressed as means, standard deviation and proportions.

## Results

A total of ninety two (92) patients who were referred for upper gastrointestinal endoscopy were included in this survey. Endoscopy findings showed characteristic GERD features in 81 individuals. Eleven individuals had other upper gastrointestinal conditions different from GERD. We therefore described nutritional status, GERD risk factors, clinical symptoms and GERD precipitating substances or food in these 81 endoscopy proven GERD patients. Our study population had a mean age of 47.32 (standard deviation 17) years, and 49 (53%) of them were males (Table 1).

**Table 1:** Age and sex proportions among GERD patients N= 92

Age groups	Male	Female
<25	06(60)	4(40)
25-34	06(35)	11(65)
35-44	07(47)	8(53)
45-54	07(37)	12(63)
55-64	8(62)	05(38)
>64	15(83)	03(17)

Mean age of 47.32, Standard deviation 17 years, Males 53%

**Figure 1:**
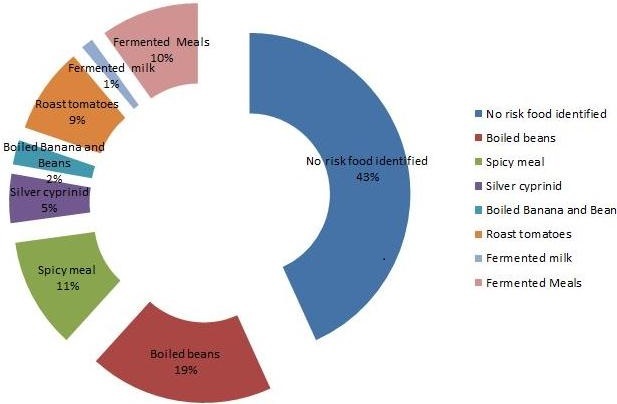
GERD symptom precipitating food substances

Most of the patients (84%) were graded as Los Angeles (LA) grade A, B or C. Only a third (31%) of patients had overweight based on their body mass indices (Table 2). More than half (56%) of the studied population recognized and could identify food substance that triggered their GERD symptoms. Identified food substances were spicy food (11%), silver cyprinid fish commonly known as dagaa in Swahili (5%), a mixture of beans and cooked green banana meal commonly known as matoke in East Africa (2%), sour milk (1%), and sour fermented meal (10%). (Figure 1 and Table 3). A substantial proportion of individuals who had GERD consumed alcohol (25%). We did not find other risks such as drugs, caffeine, mint consumption or cigarette smoking to be a common practice in this study population.

**Table 2:** Clinical characteristics-endoscopy & nutritional classification among GERD patients; N =81

**GERD LA grading**	A	19(20.7)
	B	26(28.3)
	C	23(25.0)
	D	13(14.1)
**BMI**	Weight loss	15(16.30)
	Normal weight	48(52.20)
	Overweight	29(31.50)

Most of our study participants had GERD grade A or B, (49%) ‘Normal weight’ group ad more GERD patients than the overweight group (31%)

**Table 3:** GERD symptoms provoking food and substances; N= 81

Food substances	Proportion
No risky food identified	35(43)
Perceived Risk food substances (spiced food, silver cyprinid fish cooked beans & green banana-mixture, roast tomatoes, and fermented/sour milk and Sour meals)	46(57)

More than a half of the studied population identified food substances that provoked their symptoms

**Figure 2:**
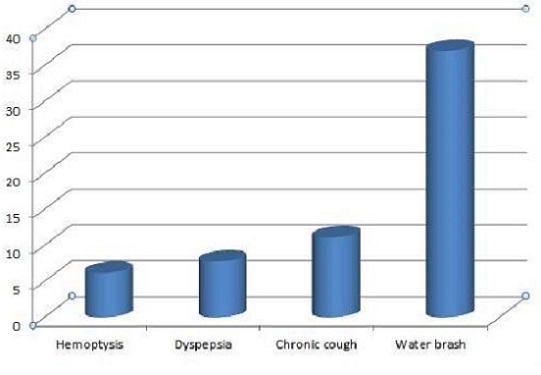
Proportion of GERD symptoms presentation in percentage

Reported oesophageal symptoms included water brash (37%) and dyspepsia (6%), chronic cough (11%) and haemoptysis (5%). Other symptoms were chronic cough (11%) and hemoptysis (5%) (Figure 2). Upper gastrointestinal endoscopy examination revealed defective lower oesophageal sphincter (‘loose lower oesophageal sphincter’) in majority (85%) of GERD patients.

## Discussion

Risk factors and precipitants of GERD symptoms have been shown to vary in different communities because of varying food, substances or life styles exposures [6, 8–10]. Generally, there is deficient evidence in the literature on GERD precipitants or risks in African's communities [2]. We therefore speculate that there haven't been enough studies or reports on these risk factors and precipitants from these communities. Partly this explains finding of our study which identified a range GERD symptoms precipitants that are not described in the literature. Life style modification by omission of GERD's precipitants forms an important component in the first line management [11, 17]; it is therefore imperative to define and identify these local precipitants if we have to optimise lifestyle changes as a management strategy.

Prevalence of obesity in developing countries is far much less than in the developed countries [18], consequently obesity is probably not an important risk factor for GERD in developing countries communities. Role of obesity in the pathophysiology of GERD have been demonstrated to be a result of an increase of intra-gastric pressure [11]. Findings of studies on obesity in GERD are contradictory and varies in different ethnic groups [7]. Some studies have found obesity not to be a risk for GERD [4] while studies on white males in developed countries have shown association of obesity and GERD [6, 7, 19]. In our study majority of our patients had normal weight or were malnourished, our findings are consistent with studies which found obesity not to be risk factor for GERD in developing world [4].

Extra oesophageal manifestation of GERD is uncommon presentation when compared to oesophageal symptoms presentation [5]. In this study, most of the patients who presented with “oesophageal symptoms” mentioned water brash and dyspepsia as their GERD symptoms; contrary to the existing literature [11], water brash which is usually infrequent presentation of GERD was found to be a common presentation in our studied population. “Extra-oesophageal” GERD manifestations were lesser and included chronic cough and haemoptysis. These findings are similar to the study conducted by Jespersen and colleagues [5].

The term “loose lower oesophageal sphincter” is used to describe patients with obvious loose defect in the lower gastro-oesophageal sphincter during endoscopy. Although the gold standard diagnostic tool for lower oesophageal sphincter incompetence is manometer [14, 15], researchers have shown correlation of lower oesophageal sphincter abnormality with endoscopy findings. In a study conducted by Falavigna and his colleagues in GERD patients, endoscopy finding of the lower part of the esophagus they described as “open cardia” showed correlation with manometry findings and Ph measurements. They consequently concluded that endoscopic appearance can be used to identify GERD [16]. Management of GERD in this group of patients who have defective lower oesophageal sphincter has been shown to be resistant to medical therapy: surgical reconstruction has therefore been regarded as the most effective therapy [20]. In resource constrained settings and where manometer are not available, endoscopy may therefore be used to timely diagnose and streamline GERD management.

## Conclusion

Our study found a range of local frequently consumed food substances identified as triggers of GERD symptoms. Water brash was found to be a frequent symptom presentation of GERD and “loose lower oesophageal” defect found to be a common upper gastrointestinal endoscopy finding. A larger study is required to confirm these findings and to correlate endoscopy and manometry findings in investigation of lower oesophageal sphincter.
