# Corrigendum: Risks, precipitants and clinical presentation of gastro-oesophageal reflux disease at the Kilimanjaro Christian Medical Centre in Tanzania

**DOI:** 10.11604/pamj.2015.20.351.6805

**Published:** 2015-04-13

**Authors:** Michael Bartholomew Mwandri, Julius Chacha Mwita, Mgaywa Gilbert Mjungu Damas Magafu, Kajiru Gad Kilonzo, Sarah Japhet Urasa

**Affiliations:** 1 University of Botswana, Private Bag 713, Gaborone, Botswana; 2 Kilimanjaro Christian Medical Centre, Tanzania P.O.Box 3010, Moshi, Tanzania

**Keywords:** Corrigendum, Pan African Medical Journal, PAMJ

## Abstract

This corrigendum corrects article “Risks, precipitants and clinical presentation of gastro-oesophageal reflux disease at the Kilimanjaro Christian Medical Centre in Tanzania”. Pan Afr Med J. 2014 Oct 1;19:119. doi: 10.11604/pamj.2014.19.119.3575.

## Corrigendum

The original version of this article contained typographical errors and incomplete authors" names. These have been corrected in both the PDF and HTML versions of the article.

1. The names of two authors were corrected. “Magafu Mgwaya” was changed to “Mgaywa Gilbert Mjungu Damas Magafu” and “Sarah Urasa” was changed to “Sarah Japhet Urasa”.

The following errors or omissions were also corrected:

**In the entire text:**

2. “gastroesophageal” was changed to “gastro-oesophageal”, “esophageal” was changed to “oesophageal” and “esophagus” was changed to “oesophagus”.

**In the abstract section:**

3. The sentence “We consecutively recruited 92 GERD patients referred for endoscopy at KCMC from March to November 2008” was corrected to “We consecutively recruited 92 GERD patients who were referred for endoscopy at KCMC from March to November 2008”.

4. “Using structured questionnaire risk factors, precipitants and symptoms of GERD were enquired. Their upper gastrointestinal endoscopic findings were as well documented.” Was corrected “By using structured questionnaire we enquired: risk factors, precipitants, symptoms of GERD and upper gastrointestinal endoscopic findings.”

**In the introduction section:**

5. “KCMC” was spelled out: “Kilimanjaro Christian Medical Centre (KCMC).”

**In the methods section:**

6. “too sick to be interviewed or measured” was changed to “too sick to be interviewed or to undertake anthropometric measurements”.

7. “research ethics at the Tumaini University” was changed to “research ethics committee at the Tumaini University”.

**In the results section:**

8. “silver cyprinid fish (5%)” was changed to “silver cyprinid fish commonly known as dagaa in Swahili (5%)”.

9. “banana meal (Matoke) (2%),” was changed to “banana meal commonly known as matoke in East Africa (2%)”.

10. The sentence “Reported oesophageal symptoms included water brash (37%) and dyspepsia (6%).Other symptoms were chronic cough (11%) and hemoptysis (5%)” was changed to “Reported oesophageal symptoms included water brash (37%), dyspepsia (6%), chronic cough (11%) and haemoptysis (5%)”.

**In the discussion section:**

11. The sentence “Life style modification by omitting exposure of food and substances that precipitate GERD symptoms forms an important” was changed to “Life style modification by omission of GERD”s precipitants forms an important component in the first line management".

12. “while on the other hand studies in white male and in developed countries communities have shown association of obesity and GERD” was changed to “while studies on white males in developed countries have shown association of obesity and GERD”.

13. “dyspepsiaas their GERD symptoms” was changed to “dyspepsia as their GERD symptoms”.

14. “loose defect on the lower gastro-esophageal” was changed to “loose defect in the lower oesophageal”.

15. “diagnostic tool for the lower esophagea” was changed to “tool for lower oesophageal sphincter”.

16. “where manometer is a rarity” was changed to “where manometers are not available”.

**In the acknowledgements section:**

17. “We acknowledge the contribution of the following for their valuable contribution to this study; Prof. George Chugulu, Dr.Chilonga Kondo, Dr. EN Swai, Prof. Kibiki, Ms. Chacha, Dr. BrigitaSwai and the Librarians of the Kilimanjaro Christian Medical Center (KCMC) Tanzania.” Was changed to “We acknowledge valuable contributions of the following persons: Prof. George Chugulu, Dr. Kondo Chilonga, Dr. E. N. Swai, Prof. G. S. Kibiki, Dr. Brigita Swai, Menyiaichi Tarimo, Rehema Minde, Tasiana Chacha, Grace Mosha and the librarians at KCMC.”

**In the authors’ contributions section:**

18. “Sarah Urasa” was changed to “Sarah Japhet Urasa”.

19. “Magafu Mgwaya- data analysis, interpretation, critical review of the article” was changed to “Mgaywa Gilbert Mjungu Damas Magafu - data analysis, interpretation and critical review of the article”.

20. “All authors read and agreed to the final version of this manuscript and equally contributed to its content and to the management of the case” was changed to “All authors read and agreed to the final version of this manuscript”.

